# Mental Health and Psychosocial Functioning Over the Lifespan of German Patients Undergoing Cardiac Catheterization for Coronary Artery Disease

**DOI:** 10.3389/fpsyt.2018.00338

**Published:** 2018-07-27

**Authors:** Anja Schaich, Anna L. Westermair, Matthias Munz, Stefan Nitsche, Bastian Willenborg, Christina Willenborg, Heribert Schunkert, Jeanette Erdmann, Ulrich Schweiger

**Affiliations:** ^1^Department of Psychiatry and Psychotherapy, University of Lübeck, Lübeck, Germany; ^2^Institute for Cardiogenetics, University of Lübeck, Lübeck, Germany; ^3^University Heart Center, University of Lübeck, Lübeck, Germany; ^4^DZHK, German Centre for Cardiovascular Research, Partner site Hamburg, Kiel, Lübeck, Germany; ^5^Deutsches Herzzentrum München (German Heart Center Munich), Fakultät für Medizin, Technische Universität München, Munich, Germany; ^6^Deutsches Zentrum für Herz-Kreislaufforschung e.V. Partner site Munich, Munich, Germany

**Keywords:** coronary artery disease, psychosocial functioning, mental disorders, mental health, prevalence rates

## Abstract

**Background:** Psychological problems are common in patients with coronary artery disease (CAD) and are associated with poor outcome. However, data on the prevalence of distinct mental disorders and their relevance to patients' functioning in daily life are scarce.

**Method:** In this retrospective study, a total of 514 German patients with CAD as diagnosed by cardiac catheterization were assessed using the Mini International Neuropsychiatric Interview 5.0.0 (M.I.N.I.) and psychosocial functioning was evaluated using the Global Assessment of Functioning (GAF) scale.

**Results:** Twenty-nine percent of the participants suffered from at least one mental disorder after the onset of their CAD (mean time since onset = 10.86 years, *SD* = 8.15). In comparison to the period before onset of CAD, elevated prevalence rates were found for severe depressive episodes, agoraphobia, dysthymia, panic disorder, and hypochondria. Predictors of mental disorders after the onset of CAD were female gender, younger age at onset of CAD as well as mental disorders and low GAF scores before onset. GAF scores decreased after the onset of CAD, recovered only partially, and were influenced by mental disorders before onset in women but not in men.

**Conclusions:** Mental disorders—especially depression and agoraphobia—are frequent in patients with CAD, with women, patients with a younger age at onset of CAD and patients with any history of mental disorders especially at risk. Regardless of whether patients meet any specific diagnostic criteria, psychosocial functioning is markedly impaired after the onset of CAD, underscoring the need for specific mental health programs for this patient population. Future research, ideally using a prospective design, is necessary to confirm these findings and to further the knowledge of prevalence rates of mental disorders and of modifiable risk factors for the development of mental disorders in patients with CAD.

## Introduction

Coronary artery disease (CAD) is a highly prevalent and impairing disease, known to be the most common cause of death in industrialized countries (1, 2). Prevalence rates of CAD are estimated at 4.8% (12-month-prevalence) (3) and 8.2% (lifetime prevalence) (4) in Germany and at 6.2% (12-month prevalence) (5) and 7.3% (6) (lifetime prevalence) in the US.

Several studies indicated that mental health problems are common in patients with CAD. Depressive symptoms in this patient population have been found in 8.2 to 35.7% of men and in 10.3 to 62.5% of women in European countries (7), and a review identified clinical significant symptoms of depression in 31 to 40% of patients with CAD and prevalence rates between 15 and 20% for major depressive disorder (8). Anxiety symptoms were found in 12.0 to 41.8% of men and 21.5 to 63.7% of women with CAD (7). Other studies found prevalence rates of anxiety disorders between 7 and 20%, with generalized anxiety disorder being most prevalent (9, 10). Research on other mental health problems in patients with CAD are scarcer, with some small studies indicating an increase in prevalence rates of e.g., binge eating disorder (11, 12). Depressive symptoms and anxiety seem to be associated with higher rehabilitation attrition rates (13) as well as worse adherence to medication (14, 15) and advised lifestyle changes (16). There is also substantial evidence, that depressive symptoms and anxiety in patients with CAD are associated with poorer prognosis, indicating a higher risk for mortality and morbidity (17–22). Comorbid mental health problems, especially depressive symptoms and anxiety are also associated with higher medical costs due to frequent hospital stays, specialist and emergency ward visits and social costs (23, 24). For these reasons, the development of adequate treatment programs for this patient group seems to be a necessary and important field. However, current data on the treatment of mental health problems in patients with CAD are inconsistent, two meta-analyses and one Cochrane review found some evidence for the effectiveness of psychological and psychiatric interventions in the reduction of depressive symptoms and anxiety but none or only small evidence for the reduction of death rates or cardiological events in patients with CAD (25–27). To date, there is little data on prevalence rates of mental disorders in German patients with CAD and little data on the association between mental health and psychosocial functioning in this patient group. The aim of the present study was to provide prevalence rates of mental disorders and psychosocial functioning over time in a large sample of German CAD patients.

## Materials and methods

### Participants

One thousand one hundred eighty-two patients with CAD (303 female, 879 male, age *M* = 62.19, *SD* = 11.13), admitted to the university hospital of Lübeck, Germany, for cardiac catheterization between February 2004 and December 2012, gave fully informed consent at the time of admission to have their data used for research purposes and to be contacted for further research purposes. Five hundred fourteen patients agreed to participate in the telephone survey when contacted by phone several years later (Md. = 5 years, IQR = 3), between March 2013 and January 2015. The exclusion criteria were lack of informed consent, inability to participate in a telephone survey (due to cognitive deficits, hearing impairment, aphasia or insufficient language skills) and exclusion of CAD during cardiac catheterization.

### Assessment of physical health

Upon admission to the hospital, participants were interviewed on cardiovascular risk factors such as arterial hypertension, diabetes, hypercholesterolemia, and smoking. Also, their BMI was determined. Cardiac function was quantified as left ventricular ejection fraction by echocardiography. All participants underwent cardiac catheterization.

### Assessment of mental health

#### Mental disorders

During the telephone survey, the Mini International Neuropsychiatric Interview (M.I.N.I. 5.0.0) was used (28, 29). The M.I.N.I. is a short, structured diagnostic interview for DSM-IV (30) and ICD-10 (31) mental disorders and has good psychometric qualities. Compared to the Composite International Diagnostic Interview (CIDI) kappa coefficient, sensitivity and specificity are good or very good for most diagnosis and inter-rater and test-retest reliability are good (32). The M.I.N.I. has been administered via telephone in previous studies (33–35). This study focused on more severe mental disorders, mild forms of mental disorders (e.g., depressive episodes with less than 7 criteria met, specific phobias, adjustment disorders) were not included in the analysis. The M.I.N.I. was administered regarding the periods before and after the onset of CAD as well as regarding the time of the interview. The onset of the CAD was defined as the year in which one of the following criteria was first met: (a) CAD symptoms (e.g., chest pain, chest tightness) not otherwise explicable, (b) cardiac catheterization showing CAD, or (c) diagnosis of a myocardial infarction. Participants were divided into four groups: continuously healthy (no mental disorder before or after onset of CAD), remitted (mental disorder before but not after onset of CAD), newly ill (mental disorder after but not before onset of CAD) and continuously ill (mental disorder before and after onset of CAD).

#### Psychosocial functioning

Psychosocial functioning was assessed using the Global Assessment of Functioning (GAF), which has been shown to possess good psychometric properties (36). According to the DSM-IV, this standard method of judgment of a patient's level of psychological, social, and occupational functioning is rated on a scale from 1 to 100 and divided into 10-point intervals for interpretation (1–10: severe impairment; 90–100 superior functioning) (37). As the GAF is scored independently of specific diagnosis and reflects the participant's level of functioning, it supplements conventional symptom- and deficit-oriented diagnoses. The GAF was scored regarding the year before onset of CAD, regarding the lowest GAF since onset of CAD and the GAF at the time of the telephone survey.

### Procedure

Participants were contacted by telephone, informed about the content and aim of the telephone survey and asked if they consented to participate. Interviews were conducted by five trained and supervised raters of the psychological and medical staff of the University of Lübeck [one psychologist, one resident physician (psychiatry), one doctoral student (medicine) and two master students (psychology)]. All interviewers were trained in the assessment and regularly supervised by the head of the department (psychiatrist). Then, the time of CAD onset was determined, and the M.I.N.I. was conducted with regard to the participant's mental health before and after the onset of CAD as well as the present. Subsequently, the GAF was scored, and participants were informed about treatment options and contact numbers if necessary and desired by the patient. In case of acute suicidality, the study protocol specified that the psychiatrist on duty should be alerted. This situation did not occur. At the end of the telephone survey, patients were offered a summary of the research findings in layman's terms, to be delivered after completion of the study; if they accepted, their email addresses were collected.

### Statistical analysis

All statistical analyses were performed using SPSS version 23.0.0.1 for Windows (SPSS Inc., USA). Cramér's V was used as a measure of effect size in χ^2^ tests. In order to analyze associations between multiple independent variables and one binary dependent variable binary logistic regression analysis were conducted. Critical differences between GAF scores were calculated at a 95% confidence level with one-sided testing, assuming validity of the equivalence hypothesis. In all other statistical analyses two-sided testing was applied. To dissect interactions in ANOVAs, we employed Hochberg's GT2 post hoc test. Given the exploratory nature of the present study, we refrained from correcting for multiple comparisons in order to prevent accumulation of type II error.

## Results

### Demographic and clinical characteristics

Of the initial 1182 patients, 207 patients were known to be already deceased, 138 patients refused to participate, 18 patients could not participate due to cognitive limitations, 26 patients could not participate because communication was not possible (e.g., due insufficient language skills, hearing impairment, or aphasia), 80 patients could not be reached within four attempts, 189 patients could not be reached due to changed contact details, and 13 patients could not be interviewed for other reasons. The final sample consisted of 514 participants (age *M* = 60.1 years, *SD* = 10.11; 125 female, age *M* = 70.7 years, *SD* = 8.31; 389 male, age *M* = 63.8 years, *SD* = 10.55). There was no difference between the final sample and the dropouts regarding sex ratio ($\chi_{\left(1\right)}^{2}$ = 1.16, *p* = 0.28) or obesity ($\chi_{\left(1\right)}^{2}$ = 2.96, *p* = 0.09). The median age of the dropout subsample (Md. = 66 years, IQR = 17) was higher than that of the participants of our study (Md. = 59 years, IQR = 15, *U* = 137063.5, *z* = −5.83, *p* < 0.001). The median age at CAD onset was 55 years (IQR = 15). Dropouts were more likely to suffer from cardiovascular risk factors at the time of initial admission, e.g., arterial hypertension ($\chi_{\left(1\right)}^{2}$ = 12.76, *p* = 0.001), diabetes ($\chi_{\left(1\right)}^{2}$ = 20.02, *p* < 0.001) and hypercholesterolemia ($\chi_{\left(1\right)}^{2}$ = 4.95, *p* = 0.03); acute or lifetime cardiac infarction ($\chi_{\left(1\right)}^{2}$ = 10.40, *p* = 0.001); and limited left ventricular ejection fraction as estimated during echocardiography ($\chi_{\left(1\right)}^{2}$ = 18.64, *p* < 0.001).

### Prevalence rates of mental disorders

To assess the prevalence rates of mental disorders over time, we computed descriptive statistics and conducted McNemar's test. The overall lifetime prevalence of mental disorders amounted to 51.4% (see Table 1), with 37.3% of participants having suffered from at least one mental disorder before the onset of their CAD and 29.0 % after onset. We found that 16.0 % of participants met the criteria for a mental disorder both before and after onset of CAD (the continuously ill), while 21.3% met the criteria for a mental disorder only before onset (the remitted) and 12.7% only after onset of CAD (the newly ill). Finally, 50% never met the criteria for a mental disorder (the continuously healthy). Higher prevalence rates after the onset of CAD were found for severe depressive episodes (McNemar χ^2^ = 15.41, *p* < 0.001), dysthymia (exact *p* = 0.041), panic disorder (exact *p* = 0.022), agoraphobia (χ^2^ = 17.36, *p* < 0.001) and hypochondria (exact *p* = 0.035). For 80.6% of participants with severe depressive episodes after onset, this was the first manifestation, as for 90.9% of participants with dysthymia, 88.0% of participants with panic disorder, 57.6% of participants with agoraphobia, and 96% of participants with hypochondria. Lower prevalence rates after the onset of CAD were found for alcohol abuse (χ^2^ = 83.27, *p* < 0.001) and alcohol dependency (χ^2^ = 18.58, *p* < 0.001).

**Table 1:** Prevalence rates of mental disorders in CAD patients.

		**Lifetime**						**Before**						**After**						**Before vs. after**		
		**Total**	**Men**	**Women**	**Men vs. women**			**Total**	**Men**	**Women**	**Men vs. women**			**Total**	**New**	**Men**	**Women**	**Men vs. women**		**Total**	**Men**	**Women**
	**n =**	**514**	**389**	**125**				**514**	**389**	**125**				**514**	**514**	**389**	**125**			**514**	**389**	**125**
		**[%]**	**[%]**	**[%]**	***p***	**OR**	**CI**	**[%]**	**[%]**	**[%]**	***p***	**OR**	**CI**	**[%]**	**[%]**	**[%]**	**[%]**	***p***	**V**	***p***	***p***	***p***
Affective disorders	Severe depressive episode^*^	94 [18.3]	71 [18.3]	23 [18.4]	n.s.	n.s.	n.s.	36 [7.4]	23 [5.9]	15 [12.0]	0.024	0.46	[0.23;0.92]	74 [14.4]	55 [11.6]	56 [14.4]	18 [14.4]	n.s.	n.s.	0.000	0.000	n.s.
	Dysthymia	22 [4.3]	17 [4.4]	5 [4.0]	n.s.	n.s.	n.s.	6 [1.2]	4 [1.0]	2 [1.6]	n.s.	n.s.	n.s.	17 [3.3]	15 [3.0]	14 [3.6]	3 [2.4]	n.s.	n.s.	0.041	0.035	n.s.
	**Total**	**107 [20.8]**	**82 [21.1]**	**25 [20.0]**	n.s.	n.s.	n.s.	**42 [8.2]**	**26 [6.7]**	**16 [12.8]**	**0.031**	**0.49**	**[0.25;0.95]**	**85 [16.5]**	**64 [13.6]**	**66 [17.0]**	**19 [15.2]**	n.s.	n.s.	**0.000**	0.000	n.s.
	Manic episode	2 [0.4]	2 [0.5]	0 [0.0]	n.s.	n.s.	n.s.	n.a.	n.a.	n.a.	n.a.	n.a.	n.a.	n.a.	n.a.	n.a.	n.a.	n.a.	n.a.	n.a.	n.a.	n.a.
	Hypomanic episode	10 [1.9]	8 [2.1]	2 [1.6]	n.s.	n.s.	n.s.	n.a.	n.a.	n.a.	n.a.	n.a.	n.a.	n.a.	n.a.	n.a.	n.a.	n.a.	n.a.	n.a.	n.a.	n.a.
Anxiety disorders	panic disorder	15 [2.9]	10 [2.6]	5 [4.0]	n.s.	n.s.	n.s.	4 [0.8]	3 [0.8]	1 [0.8]	n.s.	n.s.	n.s.	13 [2.5]	11 [2.2]	8 [2.1]	5 [4.0]	n.s.	n.s.	0.022	n.s.	n.s.
	Agoraphobia	62 [12.1]	42 [10.8]	20 [16.0]	n.s.	n.s.	n.s.	31 [6.0]	21 [5.4]	10 [8.0]	n.s.	n.s.	n.s.	57 [11.1]	31 [6.4]	38 [9.8]	19 [15.2]	n.s.	n.s.	0.000	0.012	0.001
	Social phobia	13 [2.5]	11 [2.8]	2 [1.6]	n.s.	n.s.	n.s.	11 [2.1]	9 [2.3]	2 [1.6]	n.s.	n.s.	n.s.	9 [1.8]	1 [0.2]	7 [1.8]	2 [1.6]	n.s.	n.s.	n.s.	n.s.	n.s.
	Generalized Anxiety Disorder	11 [2.1]	8 [2.1]	3 [2.4]	n.s.	n.s.	n.s.	6 [1.2]	5 [1.3]	1 [0.8]	n.s.	n.s.	n.s.	7 [1.4]	5 [1.0]	5 [1.3]	2 [1.6]	n.s.	n.s.	n.s.	n.s.	n.s.
	Obsessive-compulsive Disorder	6 [1.2]	3 [0.8]	3 [2.4]	n.s.	n.s.	n.s.	4 [0.8]	2 [0.5]	2 [1.6]	n.s.	n.s.	n.s.	4 [0.8]	2 [0.4]	1 [0.3]	3 [2.4]	0.046	0.10	n.s.	n.s.	n.s.
	**Total**	**92 [17.9]**	**65 [16.7]**	**27 [21.6]**	n.s.	n.s.	n.s.	**47 [9.2]**	**34 [8.8]**	**13 [10.4]**	n.s.	n.s.	n.s.	**78 [15.2]**	**44 [9.4]**	**53 [13.6]**	**25 [20.0]**	n.s.	n.s.	**0.000**	0.004	0.009
PTSD	Not associated with CAD	5 [1.0]	4 [1.0]	1 [0.8]	n.s.	n.s.	n.s.	n.a.	n.a.	n.a.	n.s.	n.s.	n.s.	n.a.	n.a.	n.a.	n.a.	n.a.	n.a.	n.a.	n.a.	n.a.
	Associated with CAD	6 [1.2]	4 [1.0]	2 [1.6]	n.s.	n.s.	n.s.	n.a.	n.a.	n.a.	n.s.	n.s.	n.s.	n.a.	n.a.	n.a.	n.a.	n.a.	n.a.	n.a.	n.a.	n.a.
	**Total**	**11 [2.1]**	**8 [2.1]**	**3 [2.4]**	n.s.	n.s.	n.s.	n.a.	n.a.	n.a.	n.s.	n.s.	n.s.	n.a.	n.a.	n.a.	n.a.	n.a.	n.a.	n.a.	n.a.	n.a.
Substance-related disorders	Alcohol dependency	36 [7.0]	35 [9.0]	1 [0.8]	0.002	12.30	[1.67; 90.69]	33 [6.4]	32 [8.2]	1 [0.8]	0.003	11.15	[1.51; 82.43]	8 [1.6]	3 [0.6]	8 [2.1]	0 [0.0]	n.s.	n.s.	0.000	0.000	n.s.
	Alcohol abuse	119 [23.2]	113 [29.0]	6 [4.8]	0.000	8.18	[3.5; 19.12]	94 [18.3]	90 [23.1]	4 [3.2]	0.000	9.17	[3.29; 25.51]	5 [1.0]	2 [0.5]	3 [0.8]	2 [1.6]	n.s.	n.s.	0.000	0.000	n.s.
	**Total**	**130 [25.3]**	**124 [31.9]**	**6 [4.8]**	**0.000**	**9.35**	**[4.01; 21.82]**	**127 [24.8]**	**122 [31.5]**	**5 [4.0]**	**0.000**	**11.05**	**[4.40; 27.72]**	**13 [2.5]**	**5 [1.3]**	**11 [2.8]**	**2 [1.6]**	n.s.	n.s.	**0.000**	0.000	n.s.
	Other substance abuse	4 [0.8]	3 [0.8]	1 [0.8]	n.s.	n.s.	n.s.	n.a.	n.a.	n.a.	n.a.	n.a.	n.a.	n.a.	n.a.	n.a.	n.a.	n.a.	n.a.	n.a.	n.a.	n.a.
	Other dependency e	6 [1.2]	5 [1.3]	1 [0.8]	n.s.	n.s.	n.s.	n.a.	n.a.	n.a.	n.a.	n.a.	n.a.	n.a.	n.a.	n.a.	n.a.	n.a.	n.a.	n.a.	n.a.	n.a.
	Psychotic episode	3 [0.6]	**2 [0.6]**	**1 [0.8]**	n.s.	n.s.	n.s.	n.a.	n.a.	n.a.	n.s.	n.s.	n.s.	n.a.	n.a.	n.a.	n.a.	n.a.	n.a.	n.a.	n.a.	n.a.
Eating disorders	anorexia nervosa	2 [0.4]	1 [0.3]	1 [0.8]	n.s.	n.s.	n.s.	1 [0.2]	0 [0.0]	1 [0.8]	n.s.	n.s.	n.s.	1 [0.2]	1 [0.2]	1 [0.3]	0 [0.0]	n.s.	n.s.	n.s.	n.s.	n.s.
	Bulimia nervosa	0 [0.0]	0 [0.0]	0 [0.0]	n.s.	n.s.	n.s.	0 [0.0]	0 [0.0]	0 [0.0]	n.s.	n.s.	n.s.	0 [0.0]	0 [0.0]	0 [0.0]	0 [0.0]	n.s.	n.s.	n.s.	n.s.	n.s.
	Binge eating disorder	9 [1.8]	5 [1.3]	4 [3.2]	n.s.	n.s.	n.s.	5 [1.0]	3 [0.8]	2 [1.6]	n.s.	n.s.	n.s.	6 [1.2]	4 [0.8]	2 [0.5]	4 [3.2]	0.033	0.16	n.s.	n.s.	n.s.
	**Total**	**10 [1.9]**	**6 [1.5]**	**4 [3.2]**	n.s.	n.s.	n.s.	**5 [1.0]**	**3 [0.8]**	**2 [1.6]**	n.s.	n.s.	n.s.	**7 [1.4]**	**4 [0.8]**	**3 [0.8]**	**4 [3.2]**	n.s.	n.s.	**n.s**.	n.s.	n.s.
Somato-form disorders	somatization disorder	1 [0.2]	0 [0.0]	1 [0.8]	n.s.	n.s.	n.s.	1 [0.2]	0 [0.0]	1 [0.8]	n.s.	n.s.	n.s.	0 [0.0]	0 [0.0]	0 [0.0]	0 [0.0]	n.s.	n.s.	n.s.	n.s.	n.s.
	Hypochondria	16 [3.1]	13 [3.4]	3 [2.4]	n.s.	n.s.	n.s.	4 [0.8]	4 [1.0]	0 [0.0]	n.s.	n.s.	n.s.	13 [2.5]	12 [2.4]	10 [2.6]	3 [2.4]	n.s.	n.s.	0.035	n.s.	n.s.
	**Total**	**17 [3.3]**	**13 [3.4]**	**4 [3.2]**	n.s.	n.s.	n.s.	**5 [1.0]**	**4 [1.0]**	**1 [0.8]**	n.s.	n.s.	n.s.	**13 [2.5]**	**12 [2.4]**	**10 [2.6]**	**3 [2.4]**	n.s.	n.s.	**n.s**.	n.s.	n.s.
	**Grand total**	**264 [51.4]**	**218 [56.0]**	**46 [36.8]**	**0.000**	**2.19**	**[1.45; 3.32]**	**191 [37.3]**	**158 [40.6]**	**33 [26.4]**	**0.004**	**1.92**	**[1.23; 3.01]**	**149 [29.0]**	**65 [20.2]**	**113 [29.0]**	**36 [28.8]**	n.s.	n.s.	**0.000**	0.000	n.s.

CAD, coronary artery disease; Lifetime, lifetime prevalence; Before, prevalence before onset of CAD (median duration of this period, 55 years); After, prevalence after onset of CAD (median duration = 8 years);

* *>6 depressive symptoms; PTSD, posttraumatic stress disorder; ^e^, excl. nicotine; New, incidence rates after onset of CAD; p, significance level of χ^2^ test (McNemar's χ^2^ for before-after comparison); exact p given when appropriate; OR, odds ratio (with women coded as 0 and men as 1); n.a., not assessed/not applicable; n.s., not significant; Before vs. after, comparison of prevalence rates between the period before and the period after onset of CAD*.

### Predictors of poor mental health

To identify predictors of the development of mental disorders after CAD onset, we performed binary multiple logistic regression analysis including the variables age at onset of CAD, mental health before onset of CAD, GAF in the year before onset of CAD and gender (first block), the duration of CAD defined as the period of time between the onset of CAD and the telephone interview (second block) as well as arterial hypertension, diabetes mellitus, hypercholesterolemia, nicotine consumption, body mass index, and ventricular function (third block) as predictor variables and mental health after onset of CAD as dependent variable. A model including mental health and GAF before the onset of CAD as well as age at onset of CAD and gender was a significant fit for the data ($\chi_{\left(4\right)}^{2}$ = 39.27, *p* < 0.001, R^2^ = 0.12 (Cox & Snell), = 0.17 (Nagelkerke); Hosmer-Lemeshow test: $\chi_{\left(8\right)}^{2}$ = 7.45, *p* = 0.49). Participants with any mental disorder before the onset of their CAD and female participants, respectively, were twice as likely to meet the diagnostic criteria for a mental disorder after onset as participants without mental disorder before onset and male participants (Wald (1) = 8.09, *p* = 0.004, OR = 2.18, CI = [1.27; 3.72]; Wald (1) = 4.38, *p* = 0.036, OR = 0.49, CI = [0.25;0.96]). Lower GAF before and younger age at onset of CAD were also associated with a higher risk for mental disorders after onset (Wald (1) = 7.91, *p* = 0.005, OR = 0.97, CI = [0.96;0.99] and Wald (1) = 16.79, *p* < 0.001, OR = 0.94, CI = [0.92;0 .97]). The duration of CAD; risk factors for atherosclerosis such as obesity and nicotine consumption; elements of the metabolic syndrome such as arterial hypertension, diabetes and hypercholesterolemia; and ventricular function all failed to predict mental health.

To identify predictors of severe depressive episodes after the onset of CAD, we performed binary multiple logistic regression analysis including the variables diagnosis of severe depressive episode before onset of CAD, age at onset of CAD (first block), gender, GAF in the year before onset of CAD, duration of CAD (second block) and arterial hypertension, diabetes mellitus, hypercholesterolemia, nicotine consumption, body mass index, and ventricular function (third block) as predictor variables and diagnosis of severe depressive episode after onset of CAD as dependent variable. A model including history of severe depressive episodes and age at onset of CAD was a significant fit for the data ($\chi_{\left(2\right)}^{2}$ = 31.66, *p* < 0.001, R^2^ = 0.10 (Cox & Snell), 0.17 (Nagelkerke); Hosmer-Lemeshow test: $\chi_{\left(8\right)}^{2}$ = 0.742, *p* = 0.99). Patients who had had a severe depressive episode before onset were 11 times as likely as the others to suffer from severe depressive episodes after the onset of their CAD (Wald (1) = 23.38, *p* < 0.001, OR = 11.20, CI = [4.21; 29.83]). Age at onset was negatively associated with severe depressive episodes after the onset of CAD (Wald (1) = 8.87, *p* = 0.003, OR = 0.95, CI = [0.92;0.98]). Gender; GAF before CAD onset; duration of CAD; risk factors for atherosclerosis such as obesity and nicotine consumption; elements of the metabolic syndrome such as arterial hypertension, diabetes and hypercholesterolemia; and ventricular function failed to predict severe depressive episodes after the onset of CAD.

For predicting agoraphobia after the onset of CAD, we performed binary multiple logistic regression analysis including the variables diagnosis of agoraphobia before onset of CAD, gender, age at onset of CAD, GAF in the year before onset of CAD (first block), duration of CAD (second block) and arterial hypertension, diabetes mellitus, hypercholesterolemia, nicotine consumption, body mass index, and ventricular function (third block) as predictor variables and diagnosis of agoraphobia after onset of CAD as dependent variable. A model including history of agoraphobia, GAF before onset of CAD, gender, and age at onset of CAD was a significant fit for the data ($\chi_{\left(4\right)}^{2}$ = 62.67, *p* < 0.001, R^2^ = 0.18 (Cox & Snell), 0.37 (Nagelkerke); Hosmer-Lemeshow test: $\chi_{\left(8\right)}^{2}$ = 7.70, *p* = 0.46). Patients with agoraphobia before onset were 44 times as likely as those without to suffer from agoraphobia after the onset of their CAD (Wald (1) = 34.10, *p* < 0.001, OR = 44.01, CI = [12.36; 156.74]). Women were four times as likely as men to suffer from agoraphobia after the onset of CAD (Wald (1) = 6.80, *p* = 0.009, OR = 0.26, CI = [0.09;0.72]). GAF before and age at onset of CAD were negatively associated with agoraphobia after onset (Wald (1) = 3.90, *p* = 0.048, OR = 0.98, CI = [0.95; 1.00]; Wald (1) = 5.26, *p* = 0.022, OR = 0.95, CI = [0.91;0.99]). The duration of CAD; risk factors for atherosclerosis such as obesity and nicotine consumption; elements of the metabolic syndrome such as arterial hypertension, diabetes, and hypercholesterolemia; and ventricular function failed to predict agoraphobia after the onset of CAD.

### Gender differences regarding prevalence rates of mental disorders

To examine gender differences in prevalence rates of mental disorders, we calculated χ^2^ (see Table 1). There was a gender difference in alcohol-related disorders before the onset of CAD, including both abuse ($\chi_{\left(1\right)}^{2}=$ 23.36, *p* < 0.001, OR = 9.17, CI = [3.29; 25.51]) and dependency ($\chi_{\left(1\right)}^{2}=$ 8.71, *p* = 0.003, OR = 11.15, CI = [1.51; 82.43]), resulting in differences in lifetime prevalence ($\chi_{\left(1\right)}^{2}=$ 31.53, *p* < 0.001, OR = 8.18, CI = [3.5; 19.12]; $\chi_{\left(1\right)}^{2}$ = 9.79, *p* = 0.002, OR = 12.30, CI = [167; 90.69]). Regarding the period before the onset of their CAD, men were nine times as likely as women to meet the criteria for alcohol abuse and 11 times as likely to meet the criteria for alcohol dependency. In the period after the onset of CAD, the prevalence rates of alcohol-related disorders dropped to a level similar to that found in women.

Regarding severe depressive episodes before the onset of CAD, there was a gender difference ($\chi_{\left(1\right)}^{2}$ = 5.08, *p* = 0.02, OR = 0.46, CI = [0.23;0.92]). Women were twice as likely as men to meet the criteria for severe depressive episodes in the years before their CAD manifested. However, in the years after onset, prevalence in men increased to the level found in women. Therefore, there was no gender difference in point or lifetime prevalence of severe depressive episodes.

There was a gender difference in the prevalence of binge eating disorder after the onset of CAD ($\chi_{\left(1\right)}^{2}$ = 5.92, exact *p* = 0.033, OR = 0.16, CI = [0.03;0.86]). Women were five times as likely as men to meet the criteria for binge eating disorder. The prevalence rates for the period before CAD onset showed the same trend, although not to a significant level, with women being twice as likely to suffer from binge eating disorder as men; lifetime prevalence followed a similar pattern.

Regarding prevalence of obsessive-compulsive disorder, there was a gender difference after the onset of CAD ($\chi_{\left(1\right)}^{2}$ = 5.63, exact *p* = 0.046, OR = 0.11, CI = [0.01; 1.12]). Women were 10 times more likely than men to meet the criteria for obsessive-compulsive disorder. The prevalence rates for obsessive-compulsive disorder at the other time points/periods showed the same trend, although not to a significant level, with women being two to five times as likely to suffer from obsessive compulsive disorder as men.

### Psychosocial functioning

**Figure 1:**
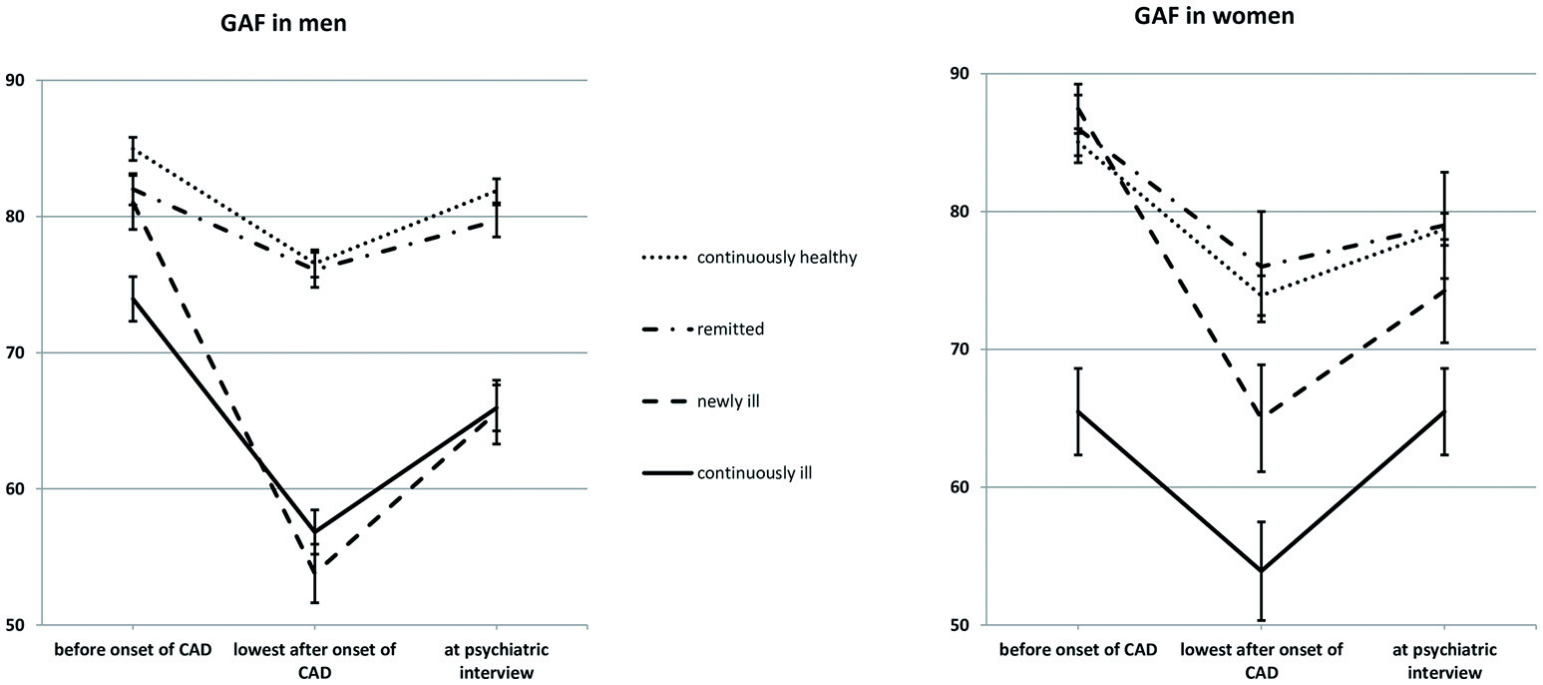
Psychosocial functioning as a function of gender and mental health. Sample size: 387 men and 125 women. Mean GAF scores are given; errors bars indicate standard errors. GAF, Global Assessment of Functioning; CAD, coronary artery disease; Continuously healthy, never met the criteria for a mental disorder before or after the onset of CAD (50.0%); Remitted, met the criteria for a mental disorder before but not after the onset of CAD (21.3%); Newly ill, met the criteria for a mental disorder after, but not before the onset of CAD (12.7%); Continuously ill, met the criteria for a mental disorder both before and after the onset of CAD (16.0%).

To examine the course of GAF over time, we conducted a three-way mixed ANOVA with repeated measures, with time as a within-subjects factor (year before onset of CAD/lowest GAF after onset/at the time of the interview), gender and mental health (continuously healthy/remitted/continually ill/newly ill) as between-subjects factors (see also Figure 1).

There was a main effect of time (Greenhouse-Geisser-corrected F_(1.77, 894.09)_ = 167.12, *p* < 0.001, $\eta_{\text{p}}^{2}$ = 0.25). The mean GAF was 82.04 (*SD* = 17.70) in the year before CAD onset and decreased to a minimum of 66.51 GAF points (*SD* = 20.32) after CAD onset [SD of Δ = 21.43, F_(1, 504)_ = 268.84, *p* < 0.001, $\eta_{\text{p}}^{2}$ = 0.35]. We found that 337 participants (65.7%) experienced significant loss of GAF, while 27 (5.3%) improved. From the onset of CAD until the interview, participants regained an average of 7.34 GAF points (*SD* = 15.61), but GAF was still decreased in comparison to the year before CAD onset [F_(1, 504)_ = 114.25, *p* < 0.001, $\eta_{\text{p}}^{2}$ = 0.19]. We found that 259 participants (50.5%) experienced significant loss of GAF between the year before onset of CAD and the interview, while 68 participants (13.3%) improved.

There was a main effect of mental health [F_(3, 502)_ = 40.91, *p* < 0.001, $\eta_{\text{p}}^{2}$ = 0.20]. Continuously ill participants scored 5.86 GAF points lower than newly ill participants (*SD* = 24.48, *p* = 0.023), who in turn scored lower than remitted participants (Δ = 8.63 GAF points, *SD* = 30.56, *p* < 0.001) as well as continuously healthy participants (Δ = 8.99 GAF points, *SD* = 30.92, *p* = 0.001). There was no difference between remitted and continuously healthy participants (Δ = 0.35 GAF points, *SD* = 34.92).

There was no main effect of gender (Δ = 1.84 GAF points, *SD* = 30.77), but there was a gender x mental health interaction (F_(3, 504)_ = 3.23, *p* = 0.022, $\eta_{\text{p}}^{2}$ = 0.02). GAF in newly ill men was similar to GAF in continuously ill men (*M* = 66.82, *SD* = 11.49 and M = 65.58, *SD* = 9.78) and lower than the GAF in remitted (M = 79.32, *SD* = 10.02) and continuously healthy men (M = 81.14,*SD* = 10.60). GAF in newly ill women was on average 75.56 GAF points (*SD* = 9.29), which was higher than that of continuously ill women (M = 65.09, *SD* = 11.10) but did not differ from GAF in remitted (M = 80.33, *SD* = 9.90) or continuously healthy women (M = 79.22, *SD* = 8.74).

## Discussion

In our sample, one in three participants met the criteria for a mental disorder after the onset of their CAD, with women, young participants and participants with a mental disorder or poor psychosocial functioning before the onset of CAD especially at risk. However, even of the participants with good mental health before the onset of CAD, one in five developed a mental disorder in the years after onset of CAD. Severe depressive episodes and dysthymia as well as agoraphobia, panic disorder and hypochondria were more than twice as prevalent after the onset of CAD, although this period was seven times shorter. Sixty to ninety percent of these affective and anxiety disorders were first manifestations. Men were half as likely as women to suffer from depression before the onset of CAD, but after onset, the probabilities equalized. Psychosocial functioning decreased after the onset of CAD and recovered only partially, even in participants who never met the criteria for a severe mental disorder. In women but not men, psychosocial functioning after the onset of CAD was influenced by mental health before the onset.

Interestingly, the prevalence of severe depressive episodes after CAD onset (14.4%, median duration of this period was 8 years) was higher than in the elderly German general population [12-month prevalence: 11.3% in women and 4.8% in men (38)] but lower than in previous studies on CAD patients (7, 8), which may be due to the fact, that our study only included more severe depressive episodes, due to overestimation in other studies because of their use of self-report measures or due to the fact that most studies assessed patients during hospitalization after an acute cardiologic event. Regarding the period before the onset of CAD, we found the well-established gender difference of 2:1 (women:men) in the prevalence rates of severe depressive episodes. After the onset of CAD, however, the prevalence in men rose to the level found in women. This is in conflict with a meta-analysis on CAD patients finding an odds ratio of 1.8:1 (women:men) (39). However, the authors themselves rated the quality of evidence as low, mainly because of lack of adjustment for confounding factors. Another reason for the difference in gender distribution in this sample could be the fact that we only included more severe depressive episodes.

The most prevalent anxiety disorder in our sample was agoraphobia, which was three to five times more frequent in our sample than in the German general population, whereas the prevalence rates of other anxiety disorder were similar (40). This is in accordance with the study by Sardinha, Araújo (12), who found agoraphobia to be highly prevalent as well as the most prevalent anxiety disorder in Brazilian CAD patients. Other studies, however, have identified GAD as the prevailing anxiety disorder in CAD patients (10–12%), with other anxiety disorders including agoraphobia being less prevalent (agoraphobia 2–4%, social phobia 3–9%, panic disorder 2–8% (9, 10)]. This discrepancy is probably not explained by phenomenological differences between study populations. More likely it is explained by the attribution of symptoms such as anxiety and avoidance to different diagnostic categories. This difference could be attributable to the use of different (versions of) psychometric instruments or intercultural differences in concepts of mental health. For example, patients in our German sample may have judged an increase in worrying as normal after a cardiac event and thus denied being overly worried. Additionally, CAD-related anxiety may not be adequately represented by any currently employed category of anxiety disorder and thus may need to be conceptualized independently in future research.

The low prevalence of post-traumatic stress disorder in our sample is consistent with data from the German general population (41). Interestingly, in the majority of participants suffering from post-traumatic stress disorder, the A1 criterion was closely associated with CAD and related medical procedures. This finding adds to the small but growing body of research on medically related post-traumatic stress disorder (42, 43) and highlights the need for research into modifiable risk factors for medically related post-traumatic stress disorder as well as routine screening of CAD patients for mental disorders followed by mental health treatment as necessary.

After the onset of CAD, binge eating disorder was twice as frequent in our sample as in the general population (44), which is in accordance with one other study published to date (12). This increased frequency of binge eating disorder could reflect selection bias as a common complication of binge eating disorder is obesity, which in turn is a risk factor for CAD, the main inclusion criterion for this study. On the other hand, CAD-related loss of mobility could leave patients with eating as one of only a few remaining emotion regulation strategies. Future research should explore this relationship between CAD and binge eating disorder in more detail, taking possible mediators such as depression into account. The gender differences in binge eating disorder found in the present study, although significant, must be interpreted with caution given the low number of cases and the type I error inflation due to multiple testing.

The finding that the prevalence of hypochondria was higher after onset, while the prevalence of somatization disorder was not, likely reflects methodological issues rather than phenomenological differences, as the DSM-IV diagnostic criteria for somatization disorder require manifestation before the age of 30. As the median onset of CAD was 55 years, CAD could not influence the development of somatization disorder. As somatization disorder has recently been reconceptualized into somatic symptom disorder and no longer holds an age criterion for first manifestation (45), this question should be addressed again in future studies.

The higher rate of mental disorders and the low psychosocial functioning after CAD onset may be due to the loss of positive reinforcements as CAD is often accompanied by functional and physical limitations which can lead to a reduction of value-based behavior (e.g., work, hobbies, sport, social activities). Another reason could be neurocognitive deterioration as a result of cerebrovascular disease, which is known to be associated with cardiovascular disease (46, 47).

Regarding risk factors for mental disorders after the onset of CAD, somatic variables were not relevant. This could be due to type II error caused by high somatic comorbidity and lack of variance in our sample, as vascular risk factors are known to be risk factors for depressive episodes (48).

The strengths of the present study are the high methodological quality of assessment of distinct mental disorders, as opposed to self-rating of mere psychological symptoms, and the consideration of all major mental disorders as opposed to only a few psychological symptoms. Furthermore, we adjusted for the confounding factor of previous mental health and supplemented diagnostic assessment with a measure of psychosocial functioning, allowing for inferences on the practical relevance of the results. The timing of assessment well after hospitalization allowed for differentiation between adaptive emotional reaction to an existential threat and psychopathology, but it also could have led to underestimation of prevalence due to selection bias caused by excess mortality in cardiac patients with severe mental disorders (49, 50). Another limitation of this study is its retrospective design, which may have led to underestimation of prevalence rates before the onset of CAD due to memory bias. A longitudinal study would be desirable. Also, milder forms of mental disorders, such as milder forms of depressive episodes and adjustment disorders were not included in this study. The risk of poor outcome in patients with CAD does seem to increase with higher severity of depressive symptomatology (19), however, even subclinical depressive symptoms have been identified to be associated with poor outcome in patients with CAD (51). This should be considered in future research on mental disorders in patients with CAD and common forms of milder mental disorders such as adjustment disorders and all kind of depressive episodes should be included. Another limitation is the lack of adjustment for socioeconomic characteristics such as socioeconomic status and marital status, which are known to influence mental health (52). The assessment of psychosocial functioning by using the GAF-score is another possible limitation of this study as data on psychometric properties of the GAF are inconsistent and more recent studies indicated weaknesses (e.g., regarding the inter-rater reliability) (53, 54). In future research alternative instruments for the assessment of psychosocial functioning such as the WHO Disability Assessment Schedule 2.0 (WHODAS 2.0) (55) should be considered. Additionally, assessment by telephone may have confounded the results due to (a) increased risk of misunderstanding because of lack of nonverbal communication and (b) selection bias as severely mentally ill patients are less likely to answer the phone. Thus, our study may underestimate the prevalence of mental disorders.

## Conclusion

Mental disorders—especially depressive and anxiety disorders—are frequent in patients with CAD, with women, young patients and patients with any history of mental disorder especially at risk. Regardless of whether patients meet any specific diagnostic criteria, psychosocial functioning is markedly impaired after the onset of CAD, underscoring the need for specific mental health programs for this patient population. Future research is necessary to explore the phenomenology of anxiety and assess somatization in CAD patients as well as search for modifiable risk factors for mental disorders in patients with somatic illnesses. Health care for CAD patients should include routine screening at regular intervals for mental disorders, followed by mental health treatment as necessary.

## Ethics statement

The study was approved by the ethics committee of Lübeck University, Germany (reference number: 04/041). Informed consent was obtained from all participants prior to inclusion in the study.

## Author contributions

BW, CW, HS, JE, and US contributed conception and design of the study. AS, AW, BW, and SN acquired the data and organized the database. AS, AW, and US performed the statistical analysis. AS and AW wrote the first draft of the manuscript. AS, AW, SN, MM, BW, CW, HS, JE, and US contributed to manuscript revision.

### Conflict of interest statement

The authors declare that the research was conducted in the absence of any commercial or financial relationships that could be construed as a potential conflict of interest.
